# Influence Mechanism of Employee Playfulness Personality on Employee Creative Deviance

**DOI:** 10.3389/fpsyg.2022.821285

**Published:** 2022-05-11

**Authors:** Qiang Liu, Zhongwei Zhao, Yiran Liu, Yu Guo, Yao He, Hao Wang

**Affiliations:** ^1^School of Economics and Management, Liaoning University of Technology, Jinzhou, China; ^2^Department of Economics and Management, Weifang University of Science and Technology, Weifang, China; ^3^School of Economics and Management, Harbin Engineering University, Harbin, China

**Keywords:** playfulness personality, creative deviance, influence mechanism, management motivation, positive impression management motivation

## Abstract

Based on the antecedent variable (playfulness personality) and the outcome variable (creative deviance) on the individual level, we introduce mediating variables (positive impression management motivation and harmonious innovation passion), integrate moderating variables (employee growth need strength and professional mission sense) to construct the conceptual model and theoretical framework of the influence mechanism of playfulness personality on creative deviance of employees. Based on the questionnaire survey data of employees in high-tech enterprises, this study adopts the nonparametric percentile Bootstrap method based on deviation correction to empirically discuss the influence mechanism of employee playfulness personality on employee creative deviance. The empirical analysis results show that employee playfulness personality has a significant positive influence on employee creative deviance. Positive impression management motivation and harmonious innovation passion partially mediate the relationships between employee playfulness personality and creative deviance. Employee growth need strength negatively moderates the relationships between positive impression management motivation and employee creative deviance. The stronger the employee growth need strength, the weaker the mediating effect of employees' playfulness personality on employee creative deviance through positive impression management motivation, and there is a moderated mediating effect.

## Introduction

With the increasingly fierce market competition, innovation occupies the market, which becomes the focus of enterprise decision-making. Managers also gradually realize that innovation can promote the rapid and effective development of enterprises, and encourage employees to innovate by means of material interest incentives, social-psychological incentives, and work incentives. However, due to the limited enterprise resources, managers will finally adopt and support a small number of innovative ideas according to the enterprise development strategy, enterprise vision and market orientation, not all innovative ideas of employees will be accepted. When innovative ideas of employees are not supported by the leaders, giving up the original ideas is a routine behavior of the employees. But it will also occur after the ideas are rejected by the leaders, employees violate the clear stop instruction and continue to adhere to their ideas for innovation privately or publicly, that is, creative deviance (Mainemelis, 2010). Employees are the main bodies of creative deviance. In the short run, creative deviance aims to produce an innovative product and refine new ideas. In the long term, the goal of creative deviance enables employees to further explore and pursue their new ideas that have been rejected and denied by the leaders, refine their new ideas in order to promote innovative behaviors and activities, enhance innovation performance, and finally improve the organizational innovation performance and organizational performance (Mainemelis, 2010; Lin et al., 2016; Wu et al., 2018).

Scholars have distinguished between creative deviance and bootlegging, the concepts of creative deviance and bootlegging are not the same. Bootlegging is initiated by employees, which is usually not known by leaders, without formal support from the organization, and is helpful to organizational innovation performance (Augsdorfer, 1996; Mainemelis, 2010; Criscuolo et al., 2014; Huang et al., 2017; Wu et al., 2018). It can be seen that there are similarities and differences between bootlegging and creative deviance. The common features between bootlegging and creative deviance are as follows: (1) they both refer to the behaviors without the regulations of organizations; and (2) they are both related to innovation. The differences between bootlegging and creative deviance are as follows: (1) bootlegging refers to that employees carry out new ideas independently and secretly, and the behaviors are hidden, while creative deviance may be carried out secretly or publicly; (2) bootlegging is when employees pursue their innovative ideas without seeking the consent of the leaders or violating the command of the leaders, while creative deviance is when employees continue to pursue new ideas in violation of the clear orders of the leaders; and (3) bootlegging includes creative deviance, but is not limited to creative deviance, and creative deviance is an extreme form of bootlegging (Augsdorfer, 1996; Mainemelis, 2010; Criscuolo et al., 2014; Huang et al., 2017; Wu et al., 2018).

Creative deviance and bootlegging can be conducive to innovation behaviors and innovation performance (Mainemelis, 2010; Criscuolo et al., 2014; Huang et al., 2017; Wu et al., 2018). The reasons that employee creative deviance is desirable lielie in (1) Creative deviance and bootlegging can be conducive to innovation behaviors. Creative deviance can enable employees to refine innovation concepts and ideas and increase the number of refined innovation concepts and ideas, generate innovative products, and enhance innovation performance. While creative deviance is constructive, creative deviance can generate positive effects (Mainemelis, 2010; Lin et al., 2016; Wu et al., 2018). (2) Creative deviance is destructive under some circumstances. The implementation of creative deviance refers to the innovation ideas with high radicality and high risk, it is difficult to convert the innovation of new products. The implementation of innovative ideas of creative deviance is clearly and explicitly rejected and denied by the leaders, which are difficult to obtain feedback information and more formal resource support, the constraints of formal resource support and the limitations of feedback information are not conducive to transform the innovation ideas into innovation products. Although creative deviance is destructive under some circumstances, once the creative deviance is successful, creative deviance can promote positive challenge psychology of employees, use saving resources to form the refined innovation solutions, simulate the independent thinking ability of employees, generate revolutionary and breakthrough innovation technologies and innovative products, and significantly improve innovation performance. Although creative deviance is destructive under some circumstances, once the creative deviance fails, employees can also increase their trial-and-error learning ability and experience, promoting experience summary and learning absorption ability. Employees can also learn and memorize from the failure, guide the future innovation behaviors and innovation activities, significantly enhance the innovation performance of employees and organizational innovation performance (Csikszentmihalyi, 1996; Benner and Tushman, 2003; Zhou, 2003; Smith and Tushman, 2005; Augsdorfer, 2008; Mainemelis, 2010; Wu et al., 2018). Although creative deviance is sometimes destructive, the benefits outweigh the risks. In general, the positive and constructive effect of creative deviance is prominent, therefore, this study advocates and carries out an empirical analysis of the creative deviance with positive and constructive effects. In addition, whether the creative deviance is desirable or not depends on the antecedent variable. At present, when the antecedent variable is positive, the antecedent variable can promote creative deviance, creative deviance is constructive and positive, the creative deviance will be strengthened and optimized. Namely, the positive antecedent variable will determine the choice preference of creative deviance and exert the rooted influential influence on the creative deviance of employees.

With reference to the domestic and abroad literature, antecedent variable of employee creative deviance consists of organizational incentive, encouraging employee innovation, positive response and feedback of the leaders, perceived underemployment, employee autonomy, forgiveness, reward rules, and approaches for employee creative deviance of the leaders, the evaluation of innovation ideas by organizations (Mainemelis, 2010; Chen et al., 2016; Lin et al., 2016; Wu et al., 2018). The empirical results of the antecedent variable of employee creative deviance are relatively few. The empirical results of employee creative deviance's formation mechanism (a driving mechanism) are relatively few.

In the serious and tense atmosphere in the enterprise, the serious and tense atmosphere of employees in the workplace is normal, which can inhibit the innovative behavior of employees to some extent (Zhu and Wang, 2019), and a positive team atmosphere can promote innovative behavior. Playfulness personality is proposed by relevant scholars, that is, working actively and enjoying, it can change the tense and serious working atmosphere and give full play to the subjective initiative of employees. Playfulness personality refers to a positive and progressive personality and trait, which is conducive. Playfulness personality positively contributes to creative thinking and productive creativity, innovative behavior, imagination, employee creativity, productivity, and innovation performance (Starbuck and Webster, 1991; Glynn and Webster, 1992; Taylor and Rogers, 2001; Yu et al., 2003; Shi et al., 2016; Wang et al., 2017). Playfulness personality is also positively conducive to work attitude, active willingness to work, happiness, new creative concepts and ideas, originality, organizational fun atmosphere (Lieberman, 1977; Glynn and Webster, 1992; Yu et al., 2003; Huang, 2006). Wang et al. (2017) further carried out an empirical analysis of the influence path and influence process of playfulness personality on employee innovation behavior and innovation performance through introducing mediating variable (job immersion) and moderating variable (career commitment), the corresponding outcome variable referred to employee innovation behavior and innovation performance. The empirical research results of introducing mediating variables and moderating variables to reveal the influence mechanism of playfulness personality on creative thinking and productive creativity, innovation behavior, imagination, employee creativity, productivity, and innovation performance are relatively few.

Creative deviance is an innovative model and way. A playfulness personality with positive and constructive attributes can promote creative deviance of employees. Employees with playfulness personality and traits will adhere to themselves and carry out creative deviance to some extent due to their enthusiasm for work after their innovative ideas are rejected. Considering the antecedent variable of creative deviance, taking the rooted influential factor as playfulness personality, the research results of the theoretical framework of the rooted influence of playfulness personality on creative deviance are lacking. The empirical research results of introducing mediating variables and moderating variables to reveal the influence mechanism of playfulness personality on creative deviance are relatively few.

Comparing with the relevant research results, this study starts from the breakthrough point of playfulness personality, sets the antecedent variable and the outcome variable as playfulness personality and creative deviance respectively, takes the mediating variables as positive impression management motivation and harmonious innovation passion, takes the moderating variables as employee growth need strength (GNS) and professional mission sense, constructs the conceptual model of the influence mechanism of employee playfulness personality on employee creative deviance from the perspective of playfulness personality, the variables in the conceptual model are all on the individual levels (employee levels). Based on the questionnaire survey data of employees in high-tech enterprises, this study adopts the nonparametric percentile Bootstrap method based on deviation correction to carry out empirical analysis of the influence mechanism of playfulness personality on employee creative deviance, empirically explores the mediating function mechanism and the moderating function mechanism through the influence path, influence effect and influence process of playfulness personality on employee creative deviance, demonstrates the mediating hub function of positive impression management motivation and harmonious innovation passion, expounds the moderating function (boundary conditions and situational factors) of employee GNS and professional mission sense, and finally extracts and forms the moderation mediating function model, deeply refining the moderation mediating effect.

The theoretical research results and empirical analysis conclusion will provide theoretical basis, theoretical significance and practical enlightenment for promoting employee creative deviance in high-tech enterprises, improving employee innovation behavior and performance, achieving the purpose of improving organizational innovation performance and enhancing organizational performance of high-tech enterprises.

## Theoretical Basis and Research Hypothesis

### Employee Playfulness Personality and Employee Creative Deviance

Lieberman (1977) first proposed that playfulness personality refers to the spontaneous personality trait. Glynn and Webster (1992) expanded the scope from children to adults based on Lieberman research results and indicated that playfulness personality had positive influence on work attitude. Yu et al. (2003) believed that playfulness personality was a trait of being active and enjoying in activities. Starbuck and Webster (1991) believed that playfulness personality could obtain pleasure from work activities and found that people with playfulness personality could better innovate. To sum up, playfulness personality is a personality trait that is active and willing to work.

Creative deviance, proposed by Mainemelis (2010), referred to the behavior of employees who violated the decision of the leaders, adhered to their own ideas, and continued to innovate after their innovative ideas were rejected by the leaders. Creative deviance is mainly divided into deviance and innovation, which is subdivided into the following four elements: (1) Leaders are aware of their innovative ideas; (2) Leaders do not approve of innovative ideas and do not support implementation; (3) Employees violate clear negative decisions; (4) Innovation creative deviance is to innovate, promote enterprise development and improve innovation performance. The creative deviance behavior is against the willingness of leaders. The results of creative deviance refer to innovative products, innovative technologies, and innovative systems.

In general, bootlegging is an innovative behavior that is carried out without the consent or even with the ignorant of leaders when the self subjectively believes that their own innovative ideas are beneficial to the enterprise (Wu et al., 2018; Wang and Zou, 2019). Both creative deviance and bootlegging are innovative behaviors beyond the regulations of enterprise. Creative deviance refers to that leaders know the innovative ideas of employees, however, in view of various considerations, without approving innovative ideas and innovative conceptions, employees continue to innovate openly or privately in violation of clear negative opinions.

Playfulness personality refers to a positive and progressive personality and trait, which is conducive. Playfulness personality is positively contributive to creative thinking and productive creativity, innovation behavior, imagination, employee creativity, productivity, innovation performance (Starbuck and Webster, 1991; Glynn and Webster, 1992; Taylor and Rogers, 2001; Yu et al., 2003; Shi et al., 2016; Wang et al., 2017). Playfulness personality is also positively conducive to work attitude, active willingness to work, happiness, new creative concepts and ideas, originality, organizational fun atmosphere (Lieberman, 1977; Glynn and Webster, 1992; Yu et al., 2003; Huang, 2006). Routine and conventional daily assembly line workflow of employee, strict customary institution rules are the obstacles to innovation behavior and innovation performance, which are destructive to revolutionary and breakthrough innovation (Mainemelis, 2010; Wei and Dang, 2018, 2020), solidification thinking and unconditional obedience to leadership decisions strangle innovative behavior of employees to some extent. The reasons why leaders deny and reject innovative ideas are that innovative ideas do not conform to the law, regulation and stipulation of enterprise innovation development, feasibility and efficiency, limited enterprise resources, and the main reason involves the limited resource problems of enterprise. Creative deviance essentially refers to breaking the above inherent mode, creative deviance plays the positive role in enterprise innovation to some extent.

Lin et al. (2016) believed that in the aspect of creative deviance, leaders conveyed the positive attitude to employees to promote their innovation performances, the purpose of leaders' positive attitude toward creative deviance was to promote employees to maintain their original work enthusiasm, role width and self-efficacy, continued to innovate. In addition to the great external influence of leaders, the optimistic and positive attitude of employees also play vital roles in the internal influence of their own personality traits. Employees with playfulness personality are more able to keep the happy attitude, who are more willing to continue to implement innovative behavior after leaders deny innovative ideas. Employees with playfulness personality have a higher concentration, a sense of prosperity, and immersion in their work (Starbuck and Webster, 1991; Glynn and Webster, 1992; Taylor and Rogers, 2001; Yu et al., 2003; Shi et al., 2016; Wang et al., 2017), who are more sensitive to the changes of the surrounding innovation environment, can better capture novel innovation ideas, strengthen innovation enthusiasm, stimulate, and derive positive emotional tone, breed innovation atmosphere (Lieberman, 1977; Glynn and Webster, 1992; Yu et al., 2003; Huang, 2006), weaken the negative opinions of leaders, adhere to their own creativity, and continue to carry out innovation behavior. Therefore, hypothesis H1 is proposed.

H1: Employees' playfulness personality has a positive influence on employee creative deviance.

### The Mediating Role of Harmonious Innovation Passion

There are relatively few research results on innovation passion by scholars at home and abroad (Lavigne et al., 2014). Fang et al. (2017) believed that innovation passion was a special form of work passion and a state of strong willingness to innovate. Vallerand et al. (2003) divided work passion into harmonious passion and compulsive passion and believed that harmonious passion was more willing to spend time and energy on some matters and affairs for internal reasons. Therefore, harmonious innovation passion belongs to work passion, which is an expression of work passion in innovation aspects. Harmonious innovation passion refers to the strong positive working state in which employees are more willing to spend time on implementing innovative behavior and more able to spend energy on innovation activities. This study carries out the mediating role of harmonious innovation passion in the relationships between employee's playfulness personality and employee creative deviance from the individual level.

Employees with playfulness personality can get the sense of pleasure from work activities and participate in innovation activities more actively (Starbuck and Webster, 1991). When innovation activities take long time, require more energy and have higher difficulty, employees with playfulness personality are more able to accept innovation challenges and innovation stressors than other employees. Playfulness personality can greatly stimulate employee curiosity, germinate innovative ideas, and then actively carry out innovative activities, it also has a high degree of concentration, participation and pleasure in innovative activities (Starbuck and Webster, 1991; Glynn and Webster, 1992; Shi et al., 2016). Playfulness personality belongs to a special personality trait, which is an optimistic and positive work mentality, the positive work mentality has initiative, positive work vitality and active work mentality can stimulate employee work willingness (Taylor and Rogers, 2001; Yu et al., 2003; Wang et al., 2017). Harmonious innovation passion is also an active willingness to work. The two sides can fit well, so as to show more active participation behavior and carry out more innovation activities. Therefore, playfulness personality can arouse harmonious innovation passion of employees, that is, playfulness personality can better show harmonious innovation passion. Therefore, hypothesis H2 is proposed.

H2: Playfulness personality can promote harmonious innovation passion.

Relevant research results show that harmonious passion promotes employees to have higher concentration (Ratelle et al., 2004) and job satisfaction (Philippe et al., 2010), and can improve creativity of employees (Qin and Zhao, 2015; Su and Lei, 2018). Wei and Zhang (2018) believed that harmonious passion could promote innovation and studied harmonious innovation passion from the team level. Harmonious innovation passion is the willingness to work when carrying out innovation activities, which promotes employees to actively participate in innovation activities, and then further stimulates innovative ideas of employees. However, not all innovative ideas of employees have been approved, the strong work willingness shown by harmonious innovation passion can enable employees to insist on the rejected ideas after their own innovative ideas are denied and continue to spend time on collecting data against the willingness of leaders to continuously improve and optimize the rejected ideas. Harmonious innovation passion urges employees to carry out and implement innovation activities, the willingness, aspiration, and desire arouse and foster employees to distribute and allocate all resources to complete innovation work, the willingness also urges employees to use enterprise innovation resources and knowledge resources to improve and supplement rejected ideas when carrying out creative deviance. Harmonious innovation passion has a positive influence on employee creative deviance. Therefore, hypothesis H3 is proposed.

H3: Harmonious innovation passion has a positive influence on creative deviance.

Based on the comprehensive hypotheses H1, H2, and H3, the mediation research hypothesis H4 is proposed:

H4: Harmonious innovation passion plays the mediating role between employees' playfulness personality and employee creative deviance.

### The Mediating Role of Positive Impression Management Motivation

Impression is the subjective understanding of the object in the human brain, the understanding will gradually form a fixed image of the object, which can usually relate to someone or thing through the image (Zhao and Yang, 2020; Guan et al., 2021). Different feelings about the appearance and interior of the object will generate different impressions (Wang et al., 2020; Qu et al., 2021). To leave good influence on each other, individuals can control their behavior and manage their impression. Impression management motivation is the psychology of hoping to be viewed positively by the other parties, according to the two ways to achieve impression management, impression management motivation is divided into two sections, namely positive impression management motivation to make the other parties look at their behavior positively, defensive impression management motivation to avoid unpopular impressions when negative situations occur (Qu et al., 2020; Wei et al., 2021). This study focuses on the mediating role of positive impression management motivation in the relationships between employee playfulness personality and creative deviance.

Making good impression on others largely stems from their own personality charm, the optimistic and positive personality traits shown by employees can help employees be treated positively (Qu et al., 2020; Wei et al., 2021). Playfulness personality can exude positive and optimistic personality charm. Positive impression management motivation is a kind of psychology that allows employees to take a positive view of themselves, compared with the tense and serious work attitude, employees prefer to the relaxed and pleasant atmosphere, the positive and optimistic work attitude is easier to leave good work impression in the psychology, mentality and mind of other employees (Qu et al., 2020; Wang et al., 2020), employees with playfulness personality have more optimistic work attitude and can be treated positively. In addition, in order to make good impression on other employees and leaders, employees will show real or untrue positive and optimistic appearance (Zhao and Yang, 2020; Guan et al., 2021). Employees with playfulness personality, together with their positive and optimistic work attitude can affect the surrounding employees to some extent, and can be more easily affirmed by leaders and other employees to obtain positive impression. To sum up, playfulness personality promotes employees to obtain positive impression, and playfulness personality can promote the generation of positive impression management motivation. Therefore, the following hypothesis H5 is proposed.

H5: Playfulness personality has a positive influence on positive impression management motivation.

The related research results of impression management motivation focus on suggestion behavior (Xiang and Long, 2013), promotional suggestion (Chen et al., 2016), citizenship behavior (Cao et al., 2016) and feedback seeking behavior (Dahling and Whitaker, 2016), and there are relatively few research results in employee innovation behavior. Zhao et al. (2019) studied the mediating mechanism of impression management motivation on innovation behavior of employees, indicating that exemplary norms could promote impression management motivation and then affected innovation behavior of employees. Tan and Liu (2017) studied the mediating mechanism of positive impression management motivation on employee creativity from the perspective of entrepreneur orientation, positive impression management motivation had positive impact on innovation behavior of employees. In face of the people with higher position and greater power, employees are more willing to create good image (Xiang and Long, 2013), when their innovative ideas of employees are denied, their innovative behavior does not get positive impression from the leader. In order to make leaders take positive view of their innovation efforts, the employees will weaken the negative opinions of leaders to some extent, they will continue to innovate during normal working hours and abnormal working hours until the innovative ideas are implemented, so as to enhance the positive impression of their leaders. Positive impression management motivation has a positive impact on employee creative deviance. Therefore, hypothesis H6 is proposed.

H6: Positive impression management motivation has a positive impact on employee creative deviance.

Based on the comprehensive hypotheses H1, H5, and H6, the mediation research hypothesis H7 is proposed:

H7: Positive impression management motivation plays the mediating role between employee playfulness personality and employee creative deviance.

### The Moderating Effect of Professional Mission Sense

Professional mission sense is a belief in seeking meaning and fun in work, and even refers to a belief that one's work is life (Duffy and Dik, 2013). Guo et al. (2019) believed that professional mission sense contained strong internal motivation to promote active behavior of employees. Gu et al. (2018) believed that professional mission sense could actively moderate the relationships between work resources and work input, which had a positive impact on work input, compared with the low professional mission sense, the high professional mission sense showed a positive sense of work input.

Relevant research results show that work involvement can promote innovative behavior of employees (Su et al., 2018). The high professional mission sense enables employees to have higher sense of work engagement and focus and be more able to carry out innovative activities. When the innovative ideas of employees are not approved, the willingness to work brought by professional mission sense makes employees unwilling to violate against the willingness of leaders. The motivation of creative deviance activities with the help of innovation passion will gradually weaken driven by their own professional mission sense, the focus and investment in their own work will weaken the negative opinions of leaders and make employees turn to their existing normal work and routine work. Clinton et al. (2017) believed that giving and endowing the strong sense professional mission senses to individuals could promote willingness of employees to work for long time. The innovation activities expected to be carried out by employees are not the results of careful consideration, innovative ideas only occur at a certain moment in some cases and circumstances (Cao and Hamori, 2020; Nurmohamed, 2020; Vogel et al., 2020), although short-term innovation passion can stimulate employee enthusiasm, subjective initiative and self-efficacy to some extent, innovation activities against the willingness of leaders need considerable innovation power and innovation persistence (Cooper et al., 2018; Jia and Liu, 2021). Employees with harmonious innovation passion also need to adhere to the innovation power, when their ideas are rejected by leaders, they need innovation power and innovation support. Employees with high professional mission senses are more willing to focus on existing routine work after innovative ideas are rejected, and they are more willing to carry out normal professional activities than spending time on the rejected innovative ideas. The professional mission sense plays a negative moderating role in the influence of harmonious innovation passion on creative deviance, professional mission sense weakens the relationships between harmonious innovation passion and creative deviance. When professional mission sense is high, the influence of harmonious innovation passion on creative deviance is low, then the employee creative deviance is low and weakened. When professional mission sense is low, the influence of harmonious innovation passion on creative deviance is high, then the employee creative deviance is high and strengthened. Therefore, hypothesis H8 is proposed.

H8: Professional mission sense plays a negative moderating role in the influence of harmonious innovation passion on creative deviance.

### The Moderating Role of Employee GNS

Hackman and Oldham (1976) first put forward the concept of employee growth need strength (employee GNS) and explained it as the individual needs and desires for growth, self-realization and accepting challenges from work. Shalley et al. (2009) indicated that employee GNS was the degree to which employees were in pursuit of dignity and self-realization at work, it was considered that the higher the employee GNS, the stronger the employee willingness to learn new knowledge and pursue better performance, and the lower the GNS, the employee motivation to pursue development opportunities at work was weak. Liu et al. (2018) confirmed that employee GNS was the psychology that employee was in pursuit of growing, developing and realizing themselves in work activities, further employee GNS could promote employee creative output.

Employees seek for promotion, salary increase and respect in the process of work, which makes employees follow the enterprise standards and stipulations in their work activities (Mitchell et al., 2019; Zou et al., 2020; Hao et al., 2021). When the own innovative ideas of employees are rejected, in order to make leaders leave good impression on their suggestions, employees will continue to study their rejected innovative ideas, realize their innovative ideas, and then prove the effectiveness and feasibility of their innovative ideas (Liu et al., 2021; Tu and Wang, 2021; Wang et al., 2021; Zhao et al., 2021), however, the related innovation research and subsequent innovation task are carried out against the willingness of leaders. In the enterprise, employees must complete the existing routine work tasks (Lu et al., 2021; Song and Chen, 2021), and creative deviance behavior is carried out after their own routine work tasks are completed. Employees with growth need strength are more willing to complete the routine work tasks assigned by the leaders at the current stage and are more willing to devote their time to obtain the positive impression of the leaders in the normal work process. Employee GNS can promote employee innovation behavior, but employees will weaken creative deviance behavior for the sake of good impression. The low-level employee GNS has insufficient motivation to accept innovation challenges and self-realization, and has no strong growth demand, they will not carry out creative deviance activities due to that their innovation ideas are rejected, they are more willing to comply with the opinions of leaders, which is not conducive to carrying out innovation activities, it is less likely to carry out creative deviance against the willingness of leaders. Employee GNS negatively moderates the relationships between positive impression management motivation and creative deviance, employee GNS weakens the influence of positive impression management motivation and creative deviance. When employee GNS is high, the influence of positive impression management motivation on creative deviance is low, then the employee creative deviance is low and weakened. When employee GNS is low, the influence of positive impression management motivation on creative deviance is high, then the employee creative deviance is high and strengthened. Therefore, the following hypothesis H9 is proposed.

H9: Employee GNS negatively moderates the relationships between positive impression management motivation and creative deviance.

According to the mediation research hypotheses (H4 and H7) and the moderated research hypotheses (H8 and H9), the moderated mediating research hypotheses (H10 and H11) are proposed:

H10: The stronger the moderating effect of professional mission sense, the weaker the mediating effect of harmonious innovation passion on the relationships between employee playfulness personality and employee creative deviance. There exists the moderated mediating roles and the moderated mediating effects.

H11: The stronger the moderating effect of employee GNS, the weaker the mediating effect of positive impression management motivation on the relationships between employee playfulness personality and employee creative deviance. There exists the moderated mediating roles and the moderated mediating effects.

**Figure 1:**
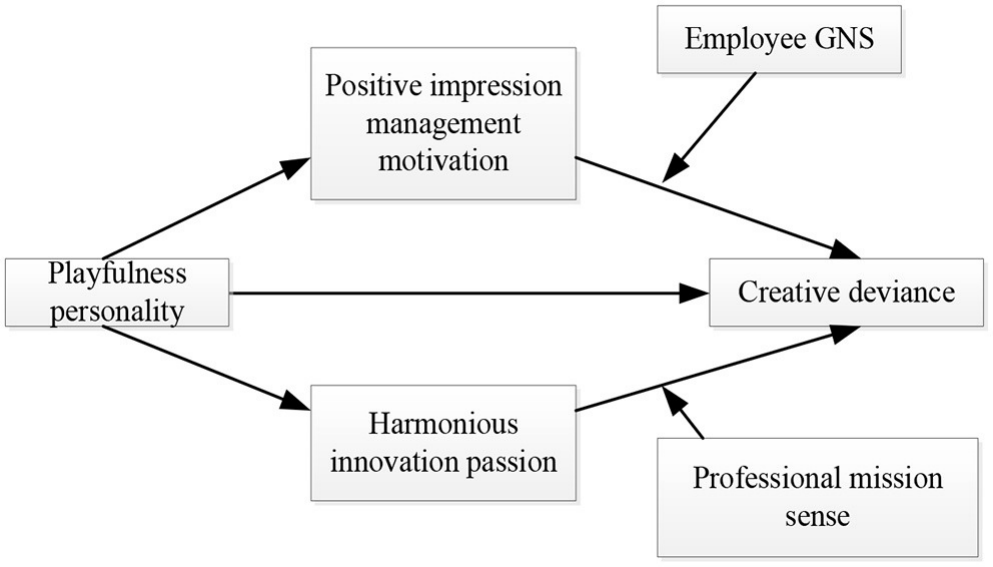
Conceptual model.

According to the above research hypotheses H1–H11, this study sets the antecedent variable and the outcome variable as playfulness personality and creative deviance respectively, takes the mediating variables as positive impression management motivation and harmonious innovation passion, takes the moderating variables as employee GNS and professional mission sense, conceptual model of the influence mechanism of employee playfulness personality on employee creative deviance is constructed, as shown in Figure 1.

## Methods

### Data Collection and Statistical Analysis

This study takes the employees of large and medium-sized high-tech enterprises in eastern China as the investigation objects. All employees are engaged in the working field of R&D and innovation. The questionnaires are distributed by means of on-site interview and on-site distribution. Researchers distribute and collect questionnaires at three different time points from January 2019 to October 2019. The first time point questionnaire investigation refers to playfulness personality, employee creative deviance and control variables (the respondents information and the respondents' enterprises information). The second time point questionnaire investigation refers to positive impression management motivation and employee GNS. The third time point questionnaire investigation refers to harmonious innovation passion and professional mission sense. The interval between each investigation is at least 1 month. At different time points, the questionnaire data collected for different variables and different control variables can accurately match each respondent.

A total of 360 questionnaires are distributed and 342 questionnaires are recovered. Excluding 53 questionnaires with incomplete questions and completely consistent answers, 289 questionnaires are effective, and the effective rates of the questionnaires are 80.28%, the relevant statistical analysis results and summary results of the control variables and demographic characteristic variables of questionnaires are shown in Table 1. In order to facilitate data analysis, the abbreviation names of playfulness personality, positive impression management motivation, employee GNS, creative deviance, harmonious innovation passion, professional mission sense as PLAY, YXDJ, GNS, CRDE, CXJQ, and SMG respectively, the abbreviation names of the variables are seen in Table 2.

**Table 1:** Statistical analysis results of questionnaire investigation data.

**Control variables and demographic characteristic variables**	**Detailed**	**Quantity**	**Proportion**
Respondents' enterprises	High-tech enterprises	289	100.00%
Regions of the respondents' enterprises	Eastern China	289	100.00%
Working fields of respondents	R&D and innovation	289	100.00%
Ages of respondents	20 years old to 30 years old	89	30.80%
	30 years old to 40 years old	100	34.60%
	40 years old to 50 years old	100	34.60%
Genders of respondents	Male	189	65.40%
	Female	100	34.60%
Education backgrounds of respondents	College degree	89	30.80%
	Bachelor degree	131	45.33%
	Graduate degree	69	23.88%
Working years of respondents	5 years to 10 years	58	20.07%
	10 years to 15 years	131	45.33%
	Over 15 years	100	34.60%
Marriage of respondents	Married	200	69.20%
	Unmarried	89	30.80%
Ages of the respondents' enterprises	5 years to 10 years	74	25.61%
	10 years to 15 years	103	35.64%
	Over 15 years	112	38.75%
Natures of the respondents' enterprises	State-owned enterprise	71	24.57%
	Private enterprise	104	35.99%
	Foreign-funded enterprises	114	39.45%
Scale of the respondents' enterprises	Large enterprises	101	34.95%
	Medium-sized enterprises	188	65.05%

**Table 2:** Abbreviation names of the variables.

**Original full name of the variables**	**Abbreviation names of the variables**
Playfulness personality	PLAY
Positive impression management motivation	YXDJ
Employee growth need strength (employee GNS)	GNS
Creative deviance	CRDE
Harmonious innovation passion	CXJQ
Professional mission sense	SMG

### Variable Measurement

Refer to relevant literatures home and abroad, this study generates the initial scales. The scales used in this study mainly refer to the more mature scales in the domestic and abroad research literatures and foundations, which are modified in combination with the research purpose and cultural situation. The questionnaires are in the form of Likert 7 scale, 1 is completely inconsistent and 7 is completely consistent. This study further tests the content accuracy, logicality and validity of the scales through two-way back translation and two-way mutual translation in order to determine the final scales. Firstly, doctors, candidates, scholars and experts in relevant fields are invited to translate the Chinese scales into the English scales, translate the English scales into the Chinese scales. Meanwhile, different doctors candidates, scholars and experts in relevant fields are invited to translate the English scales into the Chinese scales, translate the Chinese scales into the English scales. Secondly, considering cultural differences and situational factors, the experts in relevant fields and middle-senior managers of high-tech enterprises are invited to deeply check the consistency of the above two-way back translation results and two-way mutual translation results in order to avoid the language inconsistency, unclear sentences and illogical statements. The scales pass the content accuracy, logicality and validity test, the scales have relatively good internal consistency.

The measurement of playfulness personality is based on the scales of scholars Yu et al. (2003) and Huang (2006), which integrates the work characteristics of employees in high-tech enterprises, including eight items.

This study adopts the measurement scales of creative deviance of scholars Lin et al. (2016). The scales include nine items.

For the measurement of harmonious innovation passion, we select the harmonious passion part of the scales of harmonious passion and forced passion developed by scholars Sirén et al. (2016), and the innovation willingness of employees is added on this basis. Harmonious innovation passion includes seven items. The relevant results of scholars Lam et al. (2007) and Tan and Liu (2017) are used to measure the positive impression management motivation, including five items. The scales developed by scholars Dik et al. (2012) are adopted for the professional mission sense, including 12 measurement items. Employees GNS is measured by the scale of scholars Hackman and Oldham (1976) and Liu et al. (2018), including five items.

The scales and the corresponding items are in Table 3. The variables in the scales are all theoretically uni-dimensional.

**Table 3:** The scales and the corresponding items.

**The variables of the scales**	**The items of the scales**
Playfulness personality	I am easily attracted to new jobs or activities.
	When I feel stressed, I often do something fun to relax myself.
	When there is a self-breakthrough in my work, it often brings me great joy.
	I think I have a good performance at work
	At work, I can often maintain a happy mood and expression
	I have a way to make boring work fun.
	I think work is like having fun and learning from it.
	In the process of work, I will share some interesting things with my colleagues.
Positive impression management motivation	I hope to make a good impression on leaders by putting forward new ideas and skills.
	I hope to lay a good foundation for getting along with leaders in the future through positive performance.
	I hope to show my innovative ideas and views based on professional ability to leaders.
	I hope to get the attention of the leaders.
	I hope to be recognized and rewarded by the leaders.
Employee GNS	I use my imagination and creativity at work.
	I will consider all the important things in my work.
	I often exercise my ability to finish my work.
	I will create opportunities to learn new knowledge.
	I will seek for opportunities for personal growth and development.
Creative deviance	I continue to improve some of the new ideas, although the new ideas do not receive my leader's approval.
	In my work time, I often think about how to make the rejected ideas better.
	Although my leader asks me to stop developing some new ideas, I still work on these ideas.
	Besides working on ideas that are approved by my leader, I also exert effort in improving the rejected ideas by collecting information and trying again.
	I spend some of my work time in developing the ideas rejected by my leader.
	Up to this point, I still have not given up on some of the rejected ideas.
	I have improved some rejected ideas in my working hours.
	Although some ideas are stopped by the leader, I work on the improved versions of the ideas.
	Using some of my work time or resources, I keep on working on the rejected ideas.
Harmonious innovation passion	Participating in innovation activities allows me to live a variety of experiences.
	The new things that I discover in participating in innovation activities allow me to appreciate it even more.
	Participating in innovation activities allows me to have memorable experiences.
	Participating in innovation activities reflects the qualities I like about myself.
	Participating in innovation activities is in harmony with the other activities in my life.
	For me, participating in innovation activities is a passion that I still manage to control.
	I am completely taken with participating in innovation activities.
Professional mission sense	I am convinced that I am called to this kind of work I am currently engaged in.
	My work helps me achieve my life goal.
	I believe that the pressure in front of me can help me promote my career.
	The most important part of my career is to meet the needs of the works.
	I am attracted by something, which spurs me into my present work.
	In my career, changing other people's lives is my basic motivation.
	I see my career as a way to realize the meaning of life.
	My work has contributed to public wealth.
	My career is an important part of the meaning of my life.
	I often try to evaluate the benefits of my work to others.
	I have been doing my best in the present work, and achieve my belief.
	When I work, I try to make my career match the significance of life.

## Results

### Common Method Bias and Non-response Bias Test Results

This study adopts structural equation modeling methods to test the common method bias. Single factor model, two factors model, three factors model, four factors model, five factors model, and six factors model are constructed according to structural equation modeling methods. The goodness of fit indexes of single factor model, two factors model, three factors model, four factors model, five factors model, and six factors model are seen in Table 4. The goodness of fit index of single-factor model is lower than the critical value of common method bias. Furthermore, compared with the single-factor model, two factors model, three factors model, four factors model, and five factors model, the goodness of fit index of the six factors model meets the specified standard (The ratio of Chi-square to the degree of freedom is lower than the critical value of 3, TLI and CFI are higher than the critical value of 0.9, RMSEA is lower than the critical value of 0.08). Based on the six factors model, this study adds to the latent factor of the common method, and forms the six factors model including the latent factor of the common method to demonstrate the common method bias. Compared the six factors model with the six factors model including the latent factor of the common method, the change value of CFI is 0.009, the change value of TLI is 0.005, the change value of RMSEA is −0.01, all the change values are lower than the critical value 0.02, after adding to the latent factor of the common method, the goodness of fit index of six factors model has not been significantly optimized and improved. In summary, there is no serious common method bias in this study.

**Table 4:** Common method bias test results.

**Factor model**	**The corresponding factors**	**The ratio of Chi square to degree of freedom**	**CFI**	**TLI**	**RMSEA**
Single factor model	PLAY+YXDJ+GNS+CRDE+CXJQ+SMG	4.328	0.790	0.607	0.093
Two factors model	PLAY, YXDJ+CXJQ+GNS+SMG+ CRDE	3.874	0.837	0.671	0.084
Three factors model	PLAY, YXDJ+CXJQ+GNS+SMG, CRDE	3.212	0.871	0.853	0.081
Four factors model	PLAY, YXDJ+CXJQ, GNS+SMG, CRDE	3.129	0.886	0.870	0.076
Five factors model	PLAY, YXDJ, SMG, CXJQ+GNS, CRDE	3.045	0.895	0.877	0.073
Six factors model	PLAY, YXDJ, CXJQ, GNS, SMG, CRDE	2.889	0.918	0.904	0.068
Six factors model+ the latent factor of the common method	PLAY, YXDJ, CXJQ, GNS, SMG, CRDE, the latent factor of the common method	2.738	0.927	0.909	0.058

Compare the sample recovered in the early stage with the sample recovered in the later stage, this study conducts a *t*-test on the differences in the mean values of the core variables (PLAY, YXDJ, GNS, CRDE, CXJQ, and SMG). The *t*-test results show that as for the early-recovery sample and later-recovery sample, there are no significant differences among the mean values of six variables, the p values range from 0.13 to 0.25, which are higher than the critical value of 0.1. Compare the response questionnaires with non-response questionnaires, this study conducts *t*-test on the differences of the eight control variables (control variables include respondents' information and the respondents' enterprises information, namely ages of respondents, genders of respondents, education backgrounds of respondents, working years of respondents, marriages of respondents, ages of the respondents' enterprises, natures of the respondents' enterprises, scales of the respondents' enterprises) of the questionnaires. The same number of response questionnaires (40 questionnaires) and non-response questionnaires (40 questionnaires) are randomly selected. The *t*-test results show that for response questionnaires and non-response questionnaires, there are no significant differences in the control variables among the response questionnaires and non-response questionnaires, the *p*-values range from 0.23 to 0.36, which are higher than the critical value of 0.1. In summary, there is no serious non-response bias in this study.

### Reliability and Validity Test Results

After the theoretically uni-dimensional scales, we further use Mplus software to conduct confirmatory factor analysis of the scales and CFA test on each measurement model, and the output CFA results of the scales are shown in Table 5. According to the data in Table 5, the factor loading of each variable is >0.6, which is in the ideal state and within the acceptable range, with good structural validity. The SRMR of employee playfulness personality, creative deviance, positive impression management motivation, employee GNS, harmonious innovation passion and professional mission sense are within 0.08, the indicators of CFI and TLI of employee playfulness personality, creative deviance, positive impression management motivation, employee GNS, and harmonious innovation passion are >0.9, and the CFI and TLI of professional mission sense are close to 0.9, which are acceptable.

**Table 5:** Confirmatory factor analysis (structural validity test results).

**Construct**	**Factor loading**	**SRMR**	**CFI**	**TLI**
PLAY	0.680–0.851	0.047	0.929	0.901
CRDE	0.787–0.822	0.032	0.947	0.921
CXJQ	0.766–0.884	0.019	0.983	0.965
YXDJ	0.784–0.853	0.017	0.987	0.973
SMG	0.769–0.851	0.043	0.889	0.864
GNS	0.710–0.760	0.011	1.000	1.009

The combination reliability (CR) and convergence validity (AVE) are calculated according to the factor loading obtained in Table 5, the results are shown in Table 6, according to the data in Table 6, the CR values are >0.9 and the AVE values are >0.5, indicating that the scales have good convergence validity. According to the collected data, the internal consistency of the scales is tested. The Cronbach's alpha values after item deletion are smaller than the original Cronbach's alpha values, which are >0.7, CITC values are >0.5, which shows that the scales have good internal consistency, and the reliability test of the scales pass. The discriminant validity of the scales is further tested according to the Pearson correlation coefficient and AVE statistics among variable dimensions, the test results are shown in Table 7, it can be seen from the data in Table 7 that the square values of variable AVE are greater than the correlation coefficient among variables, and the scales have good discriminant validity. Moreover, this study also adopts structural equation modeling methods to determine the discriminant validity of the scales. Single-factor model, two factors model, three factors model, four factors model, five factors model, and six factors model are constructed according to structural equation modeling methods. The goodness of fit indexes of single factor model, two factors model, three factors model, four factors model, five factors model and six factors model are seen in Table 8. Compared with single-factor model, two factors model, three factors model, four factors model and five factors model, the goodness of fit index of the six factors model meets the specified standard (The ratio of the Chi-square to the degree of freedom is lower than the critical value of 3, TLI and CFI are higher than the critical value of 0.9, RMSEA is lower than the critical value of 0.08), the scales have relatively good discriminant validity.

**Table 6:** Test results of reliability and convergent validity.

**Construct**	**Cronbach's alpha**	**Cronbach's alpha if item deleted**	**CITC**	**CR**	**AVE**
PLAY	0.923	0.906–0.920	0.670–0.814	0.937	0.651
CRDE	0.946	0.938–0.942	0.772–0.827	0.955	0.701
CXJQ	0.944	0.933–0.939	0.774–0.840	0.954	0.749
YXDJ	0.916	0.892–0.905	0.746–0.806	0.937	0.748
SMG	0.959	0.955–0.957	0.754–0.835	0.964	0.692
GNS	0.853	0.817–0.829	0.646–0.689	0.895	0.631

**Table 7:** Test results of discriminant validity.

**Construct**	**AVE**	**CRDE**	**CXJQ**	**GNS**	**PLAY**	**SMG**	**YXDJ**
CRDE	0.701	**0.837**					
CXJQ	0.749	0.674	**0.865**				
GNS	0.631	0.736	0.784	**0.794**			
PLAY	0.651	0.729	0.739	0.629	**0.807**		
SMG	0.692	0.616	0.403	0.443	0.404	**0.832**	
YXDJ	0.748	0.628	0.497	0.486	0.479	0.812	**0.865**

*Diagonal bold is $\sqrt{AVE}$, and lower triangle is Pearson correlation coefficient value among variable dimensions*.

**Table 8:** Test results of discriminant validity based on structural equation modeling methods.

**Factor model**	**The corresponding factors**	**The ratio of Chi square to degree of freedom**	**CFI**	**TLI**	**RMSEA**
Single factor model	PLAY+YXDJ+GNS+CRDE+CXJQ+SMG	4.328	0.790	0.607	0.093
Two factors model	PLAY, YXDJ+CXJQ+GNS+SMG+ CRDE	3.874	0.837	0.671	0.084
Three factors model	PLAY, YXDJ+CXJQ+GNS+SMG, CRDE	3.212	0.871	0.853	0.081
Four factors model	PLAY, YXDJ+CXJQ, GNS+SMG, CRDE	3.129	0.886	0.870	0.076
Five factors model	PLAY, YXDJ, SMG, CXJQ+GNS, CRDE	3.045	0.895	0.877	0.073
Six factors model	PLAY, YXDJ, CXJQ, GNS, SMG, CRDE	2.889	0.918	0.904	0.068

### Research Hypothesis Test Results

#### Main Effect and Mediating Effect Test

This study adopts the nonparametric percentile Bootstrap method based on deviation correction, and uses the macro program of process confidence interval in SPSS software (PROCESS Procedure for SPSS Version 3.2), and sets the number of Bootstrap operation samples as 5,000, takes the confidence level and confidence interval as 95%. The test results of the main effect are seen in Table 9. It can be seen from Table 9 that the main effect (total effect) of employee playfulness personality on employee creative deviance is 0.720, the significance level of the test is *p* < 0.05, and the research hypothesis H1 is supported.

**Table 9:** Main effect test results.

**Effect**	**Estimate**	**Product of Coefficients**			**Bootstrap 5,000 times 95% CI**	
		**S.E**.	**Est./S.E**.	***p*-value**	**Lower**	**Upper**
Main effect (PLAY → CRDE)	0.720	0.051	14.196	0.000	0.614	0.815

This study adopts the nonparametric percentile Bootstrap method based on deviation correction, uses the macro program of the process confidence interval in SPSS software (PROCESS Procedure for SPSS Version 3.2) and sets the number of Bootstrap operation samples as 5,000, takes the confidence level and confidence interval as 95%. The test results of the mediating effects are seen in Tables 10–12. Table 10 test results show that the path coefficient results of the influence of employee playfulness personality on positive impression management motivation and harmonious innovation passion are 0.401 and 0.709, respectively, and the *p*-value is < 0.05, employee playfulness personality has a significant promoting effect on positive impression management motivation and harmonious innovation passion (Hypothesis H5 and hypothesis H2 are supported). The path coefficients of positive impression management motivation and harmonious innovation passion on employee creative deviance are 0.385 and 0.200, respectively, and the significance level is <0.05, positive impression management motivation and harmonious innovation passion have significant positive influences on creative deviance (Hypothesis H6 and hypothesis H3 are supported). According to the test results of direct effect, indirect effect and total effect in Table 11, the direct effect of employee playfulness personality on employee creative deviance is significant, with a value of 0.424, the mediating effects of indirect influence of employee playfulness personality on employee creative deviance through harmonious innovation passion and positive impression management motivation are 0.142 and 0.154, respectively, and the total indirect effect value is 0.296, and the corresponding confidence interval does not include 0. Table 12 shows that there is no significant difference between the two indirect mediating paths of the influence of employee playfulness personality on creative deviance, and there is no significant difference between the influence of employee playfulness personality on employee creative deviance through harmonious innovation passion and positive impression management motivation (The difference value of the indirect mediating effect is 0.013, the confidence interval includes 0, and the corresponding *p* is higher than 0.05). To sum up, the research hypotheses that H4 and H7 are supported, harmonious innovation passion plays the mediating role between employee playfulness personality and employee creative deviance, and positive impression management motivation plays the mediating role between employee playfulness personality and employee creative deviance.

**Table 10:** Test results of mediating effects.

**Dependent variables**	**Independent variables**	**Estimate**	**Product of coefficients**			**Bootstrap 5,000 times 95% CI**	
			**S.E**.	**Est./S.E**.	***p*-value**	**Lower**	**Upper**
CRDE	PLAY	0.424	0.071	5.950	0.000	0.271	0.552
	YXDJ	0.385	0.046	8.386	0.000	0.299	0.479
	CXJQ	0.200	0.070	2.875	0.004	0.077	0.349
YXDJ	PLAY	0.401	0.041	9.783	0.000	0.320	0.480
CXJQ	PLAY	0.709	0.043	16.650	0.000	0.621	0.789

**Table 11:** Test results of direct effect, indirect effect and total effect.

**Effects**	**Estimate**	**Product of coefficients**			**Bootstrap 5,000 times 95% CI**	
		**S.E**.	**Est./S.E**.	***p*-value**	**Lower**	**Upper**
Total effects	0.720	0.051	14.196	0.000	0.614	0.815
Total indirect effects	0.296	0.049	6.083	0.000	0.208	0.399
Direct effects	0.424	0.071	5.950	0.000	0.271	0.552
PLAY → YXDJ → CRDE	0.154	0.025	6.095	0.000	0.111	0.210
PLAY → CXJQ → CRDE	0.142	0.050	2.838	0.005	0.055	0.251

**Table 12:** Comparison results of mediating effects.

**Effects**	**Estimate**	**Product of coefficients**			**Bootstrap 5,000 times 95% CI**			
					**Bias corrected**		**Percentile**	
		**S.E**.	**Est./S.E**.	***p*-value**	**Lower**	**Upper**	**Lower**	**Upper**
CXJQ mediating effect	0.142	0.050	2.838	0.005	0.055	0.251	0.052	0.248
YXDJ mediating effect	0.154	0.025	6.095	0.000	0.111	0.210	0.108	0.206
Total values of mediating effects	0.296	0.049	6.083	0.000	0.208	0.399	0.206	0.396
Difference values of mediating effects	0.013	0.062	0.201	0.841	−0.119	0.129	−0.118	0.129

#### Moderating Effect Test

This study adopts the nonparametric percentile Bootstrap method based on deviation correction and uses the macro program of the process confidence interval in SPSS software (PROCESS Procedure for SPSS Version 3.2nd sets the number of Bootstrap operation samples as 5,000, and takes the confidence level and confidence interval as 95% in order to test the moderating effect of the research hypothesis. The moderating effect test results of GNS can be seen in Table 13, and the moderating effect test results of SMG can be seen in Table 14.

**Table 13:** Test results of GNS moderating effect.

**Outcome variable: CRDE**						
**Model summary**						
**R**	**R-sq**	**MSE**	***F***	**df1**	**df2**	***p***
0.8007	0.6411	0.4100	169.6935	3.0000	285.0000	0.0000
**Model**						
	**coeff**	**Se**	**T**	**p**	**LLCI**	**ULCI**
Constant	4.5194	0.0417	108.4391	0.0000	4.4374	4.6014
YXDJ	0.4245	0.0476	8.9139	0.0000	0.3308	0.5182
GNS	0.6764	0.0507	13.3517	0.0000	0.5767	0.7762
Int_1	−0.1115	0.0475	−2.3487	0.0195	−0.2050	−0.0181
**Product terms key:**						
**Int_1: YXDJ × GNS**						
**Test(s) of highest order unconditional interaction(s):**						
	**R** ^**2**^ **-chng**	***F***	**df1**	**df2**	***p***	
YXDJ*GNS	0.0069	5.5163	1.0000	285.0000	0.0195	
**Focal predict: YXDJ**						
**Mod var: GNS**						
**Conditional effects of the focal predictor at values of the moderator(s):**						
**GNS**	**Effect**	**Se**	**T**	**p**	**LLCI**	**ULCI**
−1.0754	0.5444	0.0719	7.5698	0.0000	0.4029	0.6860
−0.0754	0.4329	0.0480	9.0238	0.0000	0.3385	0.5273
0.7246	0.3437	0.0570	6.0282	0.0000	0.2315	0.4559

*Level of confidence for all confidence intervals in output: 95%*.

**Table 14:** Test results of SMG moderating effect.

**Outcome variable: CRDE**						
**Model summary**						
**R**	**R-sq**	**MSE**	***F***	**df1**	**df2**	***p***
0.7718	0.5956	0.4620	139.9419	3.0000	285.0000	0.0000
**Model**						
	**coeff**	**Se**	***T***	***p***	**LLCI**	**ULCI**
Constant	4.5010	0.0428	105.2725	0.0000	4.4169	4.5852
CXJQ	0.5179	0.0423	12.2422	0.0000	0.4346	0.6012
SMG	0.4350	0.0437	9.9440	0.0000	0.3489	0.5211
Int_1	−0.0568	0.0366	−1.5530	0.1215	−0.1289	0.0152
**Product terms key:**						
**Int_1: CXJQ × SMG**						
**Test(s) of highest order unconditional interaction(s):**						
	**R** ^**2**^ **-chng**	***F***	**df1**	**df2**	***p***	
CXJQ*SMG	0.0034	2.4117	1.0000	285.0000	0.1215	

*Level of confidence for all confidence intervals in output: 95%*.

The hierarchical regression analysis model 1 with moderating variable GNS, mediating variable YXDJ, and no product interaction term is constructed, and the hierarchical regression analysis model 2 with moderating variable GNS, mediating variable YXDJ and product interaction term GNS x YXDJ is constructed. Both hierarchical regression analysis models take CRDE as the dependent variable. The test results in Table 13 show that the path coefficient of the product interaction term in the hierarchical regression analysis model 2 is −0.1115, the *p*-value is 0.0195, the *p*-value is < 0.05, the corresponding confidence interval is [−0.2050, −0.0181], the confidence interval does not include 0, and the product interaction term has a significant negative influence. Hierarchical regression analysis model 2 is compared with hierarchical regression analysis model 1, the *R*^2^ change (*R*^2^-chng) value of goodness of fit index is 0.0069, *F*-value is 5.5163, *p*-value is 0.0195, *p* < 0.05, under the conditional action of low-level, medium-level and high-level moderating variables, the values of moderating effect are 0.5444, 0.4329, and 0.3437, *p*-values are 0.000, *p* < 0.05, and the corresponding confidence intervals [0.4029, 0.6860], [0.3385, 0.5273], [0.2315, 0.4559], and the confidence intervals do not include 0. To sum up, the research hypothesis of GNS moderating effect is supported by H9, and employee GNS negatively moderates the relationships between positive impression management motivation and creative deviance.

The hierarchical regression analysis model 3 with moderating variable SMG, mediating variable CXJQ and no product interaction term is constructed, and the hierarchical regression analysis model 4 with moderating variable SMG, mediating variable CXJQ, and product interaction term SMG × CXJQ is constructed. Both hierarchical regression analysis models take CRDE as the dependent variable. The test results in Table 14 show that path coefficient of product interaction term in the hierarchical regression analysis model 4 is −0.0568, the *p*-value is 0.1215, the *p*-value is higher than the specified significance level of 0.05, the corresponding confidence interval is [−0.1289, 0.0152], the confidence interval includes 0, and the product interaction term has no significant negative influence. Hierarchical regression analysis model 4 is compared with hierarchical regression analysis model 3, the *R*^2^ change value (*R*^2^-chng) of the goodness of fit index is 0.0034, *F*-value is 2.4117, *p*-value is 0.1215, and the significance level is *p* > 0.05. To sum up, the research hypothesis of SMG moderating effect H8 is not supported, and professional mission sense does not have a negative moderating effect on the influence of harmonious innovation passion on employee creative deviance.

**Figure 2:**
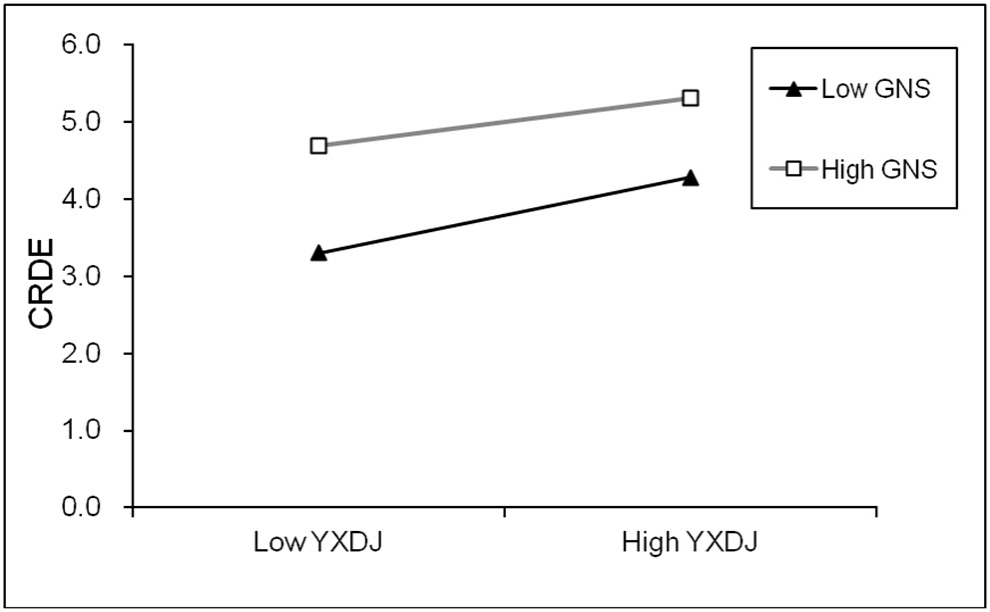
Moderating and interaction effect figure.

This study further draws the moderating and interaction effect figure according to the moderating and interaction effect drawing steps and the key analysis steps of simple slope (Aiken and West, 1991), the moderating and interaction effect figure of GNS is seen in Figure 2, the corresponding data of moderating and interaction effect figure are seen in Table 15. The mean value of GNS plus 1SD represents high GNS (GNS mean value + 1SD), the mean value of GNS minus 1SD represents low GNS (GNS mean value - 1SD), the simple slope results indicate that with GNS from high to low, the positive effect of positive impression management motivation on employee creative deviance increases gradually, the higher GNS, the lower the promoting effect of positive impression management motivation on employee creative deviance. When GNS is low, the effect of positive impression management motivation on employee creative deviance is high (effect value is 0.5444, *t*-value is 7.5698, *p* < 0.001), the relationships between positive impression management motivation and creative deviance are strengthened. When GNS is high, the effect of positive impression management motivation on employee creative deviance is low (effect value is 0.3437, *t*-value is 6.0282, *p* < 0.001), the relationships between positive impression management motivation and creative deviance are weakened.

**Table 15:** The corresponding data of moderating and interaction effect figure.

**CRDE**	**Low YXDJ**	**High YXDJ**
Low GNS	3.304	4.284
High GNS	4.701	5.320

#### The Moderated Mediating Effect Test

This study further adopts the nonparametric percentile Bootstrap method based on deviation correction, and uses the macro program of process confidence interval in SPSS software (PROCESS Procedure for SPSS Version 3.2), and sets the number of bootstrap operation samples as 5,000, takes the confidence level and confidence interval as 95%. The test results of the moderated mediating effect of research hypothesis H11 is seen in Table 16, the moderated mediating effect model is composed of the moderating variable employee GNS, the mediating variable positive impression management motivation, the antecedent variable playfulness personality, and the outcome variable creative deviance, the *R*^2^ value of each structural model is >0.19, and the model fitting effect is relatively good. The overall moderated mediating effect index test result is −0.0446, the confidence intervals BootLLCI and BootULCI are −0.078 and −0.0136 respectively, the left and right limits of the confidence interval are less than 0, and the confidence interval does not include 0, under the influence of low-level, medium level, and high-level moderating variables employee CNS, the mediating effect values of positive impression management motivation on the relationships between employee playfulness personality and creative deviance are 0.1696, 0.1313, and 0.0930 respectively, the corresponding confidence intervals are [0.1120, 0.2311], [0.0909, 0.1782], [0.0515, 0.1366], and the confidence intervals do not include 0, under the influence of low-level and high-level moderating variable employee GNS, there is a significant difference in the mediating effect of positive impression management motivation (The difference value of mediating effect is −0.0767, the confidence interval corresponding to the difference value of mediating effect is [−0.1342, −0.0234], and the confidence interval does not include 0). To sum up, when employee GNS is weak, the mediating effect of positive management motivation on the relationships between employee playfulness personality and employee creative deviance is strong, the weaker the employee GNS, the stronger the mediating effect of playfulness personality on employee creative deviance through positive impression management motivation, and the moderated mediating effect is significant, the research hypothesis H11 is supported.

**Table 16:** The moderation mediating effect test results.

**Outcome variable: YXDJ**						
**Model summary**						
**R**	**R-sq**	**MSE**	***F***	**df1**	**df2**	***p***
0.4746	0.2252	0.6395	83.4186	1.0000	287.0000	0.0000
Model						
	**coeff**	**Se**	***t***	***p***	**LLCI**	**ULCI**
Constant	−1.4755	0.168	−8.7692	0.0000	−1.8067	−1.1443
PLAY	0.4012	0.0439	9.1334	0.0000	0.3147	0.4876
**Outcome variable: CRDE**						
**Model summary**						
**R**	**R-sq**	**MSE**	***F***	**df1**	**df2**	***p***
0.8469	0.7173	0.3242	180.1091	4.0000	284.0000	0.0000
**Model**						
	**coeff**	**Se**	***t***	***p***	**LLCI**	**ULCI**
Constant	3.1932	0.1561	20.4567	0.0000	2.8859	3.5004
PLAY	0.3606	0.0412	8.746	0.0000	0.2794	0.4417
YXDJ	0.3273	0.0438	7.4757	0.0000	0.2411	0.4134
GNS	0.4470	0.0521	8.5741	0.0000	0.3444	0.5496
Int_1	−0.1112	0.0422	−0.6335	0.0089	−0.1943	−0.0281
**Product terms key:**						
**Int_1: YXDJ × GNS**						
**Test(s) of highest order unconditional interaction(s):**						
	**R** ^**2**^ **-chng**	***F***	**df1**	**df2**	***p***	
YXDJ*GNS	0.0069	6.9355	1.0000	284.0000	0.0089	
**Focal predict: YXDJ**						
**Mod var: GNS**						
**Conditional effects of the focal predictor at values of the moderator(s):**						
**GNS**	**Effect**	**se**	***t***	***p***	**LLCI**	**ULCI**
−0.8596	0.4228	0.0585	7.2281	0.0000	0.3077	0.5380
0.0000	0.3273	0.0438	7.4757	0.0000	0.2411	0.4134
0.8596	0.2317	0.0552	4.1993	0.0000	0.1231	0.3403
**Direct effect of PLAY on CRDE**						
**Effect**	**se**	***t***	***p***	**LLCI**	**ULCI**	
0.3606	0.0412	8.7463	0.0000	0.2794	0.4417	
**Conditional indirect effects of PLAY on CRED:**						
**Indirect effect:**						
**PLAY -> YXDJ -> CRDE**						
**GNS**	**Effect**	**BootSE**	**BootLLCI**	**BootULCI**		
−0.8596	0.1696	0.0306	0.1120	0.2311		
0.0000	0.1313	0.0225	0.0909	0.1782		
0.8596	0.0930	0.0216	0.0515	0.1366		
**Index of moderated mediation**						
	**Index**	**BootSE**	**BootLLCI**	**BootUCI**		
GNS	−0.0446	0.0163	−0.0780	−0.0136		
**Pairwise contrasts between conditional indirect effects (Effect1 minus Effect2)**						
**Effect1**	**Effect2**	**Contrast**	**BootSE**	**BootLLCI**	**BootULCI**	
0.1313	0.1696	−0.0383	0.0140	−0.0671	−0.0117	
0.0930	0.1696	−0.0767	0.0280	−0.1342	−0.0234	
0.0930	0.1313	−0.0383	0.0140	−0.0671	−0.0117	

*Level of confidence for all confidence intervals in output: 95%. Number of Bootstrap samples for percentile Bootstrap confidence intervals: 5000. GNS values in conditional tables are the mean and +/- SD from the mean*.

Due to that the research hypothesis H8 of SMG moderating effect is not supported, and the professional mission sense does not have negative moderating effect on the influence of harmonious innovation passion on creative deviance, it is not necessary to test the moderated mediating effect of research hypothesis H10, and research hypothesis H10 is not supported.

#### Research Hypothesis Test Summary

According to the above data analysis and research hypothesis test, the summary of specific research hypothesis test results are obtained, as shown in Tables 17, 18.

**Table 17:** Research hypothesis test results summary 1.

**Dependent variables**	**Independent variables**	**Research hypothesis**	**Estimate**	**S.E**.	***p***	**LLCI**	**ULCI**	**Test results**
CRDE	PLAY	H1	0.720	0.051	0.000	0.614	0.815	Support
YXDJ	PLAY	H5	0.401	0.041	0.000	0.320	0.480	Support
CXJQ	PLAY	H2	0.709	0.043	0.000	0.621	0.789	Support
CRDE	PLAY		0.424	0.071	0.000	0.271	0.552	Support
	YXDJ	H6	0.385	0.046	0.000	0.299	0.479	Support
	CXJQ	H3	0.200	0.070	0.004	0.077	0.349	Support

**Table 18:** Research hypothesis test results summary 2.

**Research hypothesis contents**	**Research hypothesis codes**	**Estimate (main indicators)**	**S.E**.	***p***	**LLCI**	**ULCI**	**Test results**
Indirect mediating effect PLAY → YXDJ → CRDE	H7	0.154	0.025	0.000	0.111	0.210	Support
Indirect mediating effect PLAY → CXJQ → CRDE	H4	0.142	0.050	0.005	0.055	0.251	Support
Moderating effect of GNS	H9	−0.1115	0.0475	0.0195	−0.2050	−0.0181	Support
Moderating effect of SMG	H8	−0.0568	0.0366	0.1215	−0.1289	0.0152	Nonsupport
The moderated mediating effect (GNS)	H11	−0.0446	0.0163		−0.078	−0.0136	Support
The moderated mediating effect (SMG)	H10						Nonsupport

## Conclusion and Discussion

### Research Conclusion

This study introduces the mediating variables (positive impression management motivation and harmonious innovation passion), and integrates the moderating variables (employee growth needs strength and professional mission sense) to construct the conceptual model of the influence mechanism of playfulness personality on employee creative deviance. This study adopts the non-parametric percentile Bootstrap method based on deviation correction to carry out an empirical analysis of the influence mechanism of employee playfulness personality on employee creative deviance. The main results of influence mechanism of employee playfulness personality on employee creative deviance are seen as follows:

1. Main effect. The main effect of employee playfulness personality on employee creative deviance is significant, and employee playfulness personality can significantly promote employee creative deviance.
2. Mediating effect. Harmonious innovation passion and positive impression management motivation play partial mediating roles in the relationships between employee playfulness personality and creative deviance, employee playfulness personality has an indirect influence on employee creative deviance by affecting harmonious innovation passion and positive impression management motivation. On one hand, playfulness personality is a personality trait that enables employees to show positive willingness to participate in innovation activities, under the action of strong harmonious innovation passion, the willingness to participate can promote the transformation of employee innovative ideas. On the other hand, the personality trait of being active and willing to work is easy to obtain good impression in the psychology, mentality, mind of leaders and other employees, and put emphasis on controlling impression of each other. After the rejection of their own innovation concepts, the positive and optimistic personality trait is beneficial to activate the motivation and psychology of obtaining positive impression, urges employees to continue to implement innovation activities against the willingness of leaders. When carrying out creative deviance, they will take the initiative to spend more time and energy, make use of their personal resources and enterprise resources, and continuously improve and optimize their own innovative ideas, framework and design thinking, achieve innovation recognition and innovation performance.
3. Moderating effect. Employee GNS has an influence on creative deviance, and employee GNS significantly negatively moderates the influence of positive impression management motivation on creative deviance. Weaker employee GNS can moderate the influence of positive impression motivation on creative deviance.
4. The moderated mediating effect. Driven by moderating variable employee GNS, the mediating effect of positive impression management motivation between the relationships of employee playfulness personality and employee creative deviance is gradually weakened. As the buffer variable and boundary condition, employee GNS weakens the mediating link and bridge role of positive impression management motivation in the relationships between employee playfulness personality and employee creative deviance. Compared with employee creative deviance to obtain positive impression, employees with high growth need strength motivation pay more attention to the existing routine and standardization work, and focus on participating in normal innovation activities in the existing work field, so as to inhibit the emergence of creative deviance behavior.

### Theoretical Significance

1. This study sets the perspective of employee playfulness personality, takes the antecedent variable as playfulness personality in order to deeply explore the antecedent variable of employee creative deviance, demonstrate the rooted influential variable of creative deviance, verify the formation mechanism and driving mechanism of employee creative deviance. The main effect indicates that playfulness personality has a positive influence on constructive creative deviance, which exerts significantly positive rooted effect. The research results expound and enrich the theoretical framework and conceptual model of the antecedent variable of creative deviance on the individual level, echo, respond, advocate the theoretical research achievements of creative deviance with positive and constructive effect, increase the theoretical research basis and foundation of the outcome variable of playfulness personality, enhance the applicable scope of playfulness personality and the influence of individual positive personality trait on innovation aspects and new innovation models.
2. This study selects mediating variables as positive impression management motivation and harmonious innovation passion, improves and enriches the conceptual model and theoretical analysis framework of the influence mechanism of playfulness personality on employee creative deviance from the perspectives of mediating variables (positive impression management motivation and harmonious innovation passion). Furthermore, we adopt the non-parametric percentile Bootstrap method based on deviation correction, implements Bootstrapping operation with a view to empirically exploring the mediating function mechanism of the influence process and influence path of playfulness personality on employee creative deviance, deeply refining the positive mediating effects of the relationships between playfulness personality and employee creative deviance, enriching the theoretical research basis, foundation and relevant results of playfulness personality and employee creative deviance, enhancing the applicability and effectiveness of the mediating variables (positive impression management motivation and harmonious innovation passion) of the relationships between playfulness personality and employee creative deviance.
3. This study selects moderating variables (employee growth need strength and professional mission sense) as boundary conditions and situational factors, improves and enriches the conceptual model and theoretical analysis framework of the influence mechanism of playfulness personality on employee creative deviance from the perspectives of moderating variables (employee growth need strength and professional mission sense). Furthermore, we adopt the nonparametric percentile Bootstrap method based on deviation correction, implements Bootstrapping operation with a view to empirically exploring the moderating function mechanism of the influence process and influence path of mediating variables (positive impression management motivation and harmonious innovation passion) on employee creative deviance, deeply refining the positive moderating effects of the relationships among mediating variables (positive impression management motivation and harmonious innovation passion) and employee creative deviance, enriching the theoretical research basis, foundation and relevant results of mediating variables (positive impression management motivation and harmonious innovation passion) and employee creative deviance, enhancing the applicability operability, feasibility of the moderating variables of employee growth need strength and professional mission sense of the relationships among mediating variables and employee creative deviance.
4. This study fuses and integrates antecedent variable (playfulness personality), mediating variables(positive impression management motivation and harmonious innovation passion), moderating variables (employee growth need strength and professional mission sense) and outcome variable(creative deviance) into the same conceptual model and theoretical analysis framework, forms the moderation mediating function model, generates the moderation mediating effect, improves the moderation mediating effect function, derives and evolves the influence mechanism of playfulness personality on employee creative deviance with completeness, systematicness and commanding unity, demonstrates the function direction, function strength and significance level of mediating effects and mediating function under the moderating variables, and enriches the empirical research results of boundary conditions and situational factors of playfulness personality and creative deviance in the aspects of organizational personality trait, organizational psychology, organizational cognition and organizational behavior.

### Practical Enlightenment

This study empirically studies the influence mechanism of employee playfulness personality on employee creative deviance of employees, in order to explore the realization path of creative deviance. High-tech enterprises are facing with the resource allocation and distribution problems, and the limited enterprise resources cannot support the innovative ideas of all employees. However, innovation is an indispensable driving force for the rapid development of enterprise, therefore, creative deviance of employees can alleviate the contradictions between resource allocation and employee innovation to some extent, and help enterprises improve innovation performance. Managers should put emphasis on the attitude toward creative deviance behavior of employees, timely show their expectations for innovation results, appropriately recognize innovative ideas, design thinking and framework of employees, pay attention to the boundary conditions of professional mission sense and growth need strength of employees, identify the direction, intensity and mode of professional mission sense and growth need strength of employees, encourage employees to spend their time perfecting innovative ideas after their ideas are rejected. Meanwhile, managers should also pay attention to work motivation of employees, stimulate employee innovation passion, promote harmonious innovation passion and positive impression management motivation of employees, encourage employees to implement meaningful innovation behavior, promote employees to have clear understanding of creative deviance results, and provide long-term innovation power for creative deviance. In addition, when recruiting employees, managers should focus on the employees with playfulness personality traits, create playful, fun and vibrant atmosphere for the creative team. Managers cannot blindly reject and deny the innovative ideas, framework ideology, innovation approaches and channels, design thinking, carefully consider the feasibility and operability of innovative opinions, and continue to improve and optimize creative deviance behaviors and activities, so as to stimulate the innovation passion and work passion of the whole creative team.

### Research Deficiency and Prospect

The sample is limited to the field of high-tech enterprises, which has some limitations. Further research should pay attention to the diversity of sample selection, select samples in different industries and departments, expand sample size and enhance sample diversity. In addition, this study only studies creative deviance from the individual level of employees, however, creative deviance is also affected by other levels such as leadership attitude and the overall atmosphere of creative team. In the further research, it is necessary to further adopt the cross-level analysis method to carry out empirical analysis of employee creative deviance.

## Data Availability Statement

The original contributions presented in the study are included in the article/supplementary material, further inquiries can be directed to the corresponding author.

## Ethics Statement

Ethical review and approval was not required for the study on human participants in accordance with the local legislation and institutional requirements. Written informed consent for participation was not required for this study in accordance with the national legislation and the institutional requirements.

## Author Contributions

QL: data analyses and writing of the manuscript. ZZ: critical review of the manuscript. YL, YG, YH, and HW: formal analysis. All authors contributed to the article and approved the submitted version.

## Funding

This research was funded by Research Base of Science and Technology Innovation Think Tank of Liaoning Province (Research Base of High Quality Development of Equipment Manufacturing Industry, NO.09), 2022 Economic and Social Development Research Project of Liaoning Province (20221s1qnwzzkt-011), 2019 Science Research Fund of Department of Education of Liaoning Province (JQW201915402), Humanities and Social Science Project of Shandong Province (2021-ZXCY-16), and Social Science Planning Project of Shandong Province (21CPYJ21).

## Conflict of Interest

The authors declare that the research was conducted in the absence of any commercial or financial relationships that could be construed as a potential conflict of interest.

## Publisher's Note

All claims expressed in this article are solely those of the authors and do not necessarily represent those of their affiliated organizations, or those of the publisher, the editors and the reviewers. Any product that may be evaluated in this article, or claim that may be made by its manufacturer, is not guaranteed or endorsed by the publisher.
